# Assessment of a Human Cadaver Model for Training Emergency Medicine Residents in the Ultrasound Diagnosis of Pneumothorax

**DOI:** 10.1155/2014/724050

**Published:** 2014-03-25

**Authors:** Srikar Adhikari, Wesley Zeger, Michael Wadman, Richard Walker, Carol Lomneth

**Affiliations:** ^1^Department of Emergency Medicine, University of Arizona Medical Center, P.O. Box 245057, Tucson, AZ 85724, USA; ^2^Department of Emergency Medicine, University of Nebraska Medical Center, 981150 Nebraska Medical Center, Omaha, NE 68198, USA; ^3^Department of Genetics, Cell Biology, and Anatomy, University of Nebraska College of Medicine, 981150 Nebraska Medical Center, Omaha, NE 68198, USA

## Abstract

*Objectives*. To assess a human cadaver model for training emergency medicine residents in the ultrasound diagnosis of pneumothorax. *Methods*. Single-blinded observational study using a human cadaveric model at an academic medical center. Three lightly embalmed cadavers were used to create three “normal lungs” and three lungs modeling a “pneumothorax.” The residents were blinded to the side and number of pneumothoraces, as well as to each other's findings. Each resident performed an ultrasound examination on all six lung models during ventilation of cadavers. They were evaluated on their ability to identify the presence or absence of the sliding-lung sign and seashore sign. *Results*. A total of 84 ultrasound examinations (42-“normal lung,” 42-“pneumothorax”) were performed. A sliding-lung sign was accurately identified in 39 scans, and the seashore sign was accurately identified in 34 scans. The sensitivity and specificity for the sliding-lung sign were 93% (95% CI, 85–100%) and 90% (95% CI, 81–99%), respectively. The sensitivity and specificity for the seashore sign were 80% (95% CI, 68–92%) and 83% (95% CI, 72–94%), respectively. *Conclusions*. Lightly embalmed human cadavers may provide an excellent model for mimicking the sonographic appearance of pneumothorax.

## 1. Introduction

Point-of-care ultrasound is increasingly used in the emergency department (ED) and critical care settings for the timely and accurate diagnosis of many acute conditions [1]. Early detection of a pneumothorax with bedside ultrasound can be life-saving in critically ill patients. Added to the FAST (focused assessment with sonography in trauma) examination, thoracic ultrasound can assist in the rapid assessment of trauma patients for pneumothorax and expedite management [2]. The use of ultrasound (brightness mode (B-mode) and motion mode (M-mode)) for the assessment of pneumothorax has been extensively studied in the ED and other acute care settings [3–5]. In a recent meta-analysis, the estimates for emergency ultrasound sensitivity and specificity for diagnosing pneumothorax were 90% and 98%, respectively [6].

The to-and-fro sliding of visceral and parietal pleura past each other in a normal lung on B-mode imaging is referred to as the sliding-lung sign. Comet tails are linear reverberation artifacts that originate from the visceral pleura and the presence of comet tail artifacts is a normal finding on B-mode ultrasound. In a normal lung, M-mode imaging demonstrates horizontal waves from the motionless layers of the chest above the pleural line and a granular or sandy pattern below the pleural line resembling waves crashing onto the sand which is called the seashore sign [7]. Several sonographic findings have been described to diagnose pneumothorax such as absence of sliding-lung sign and comet tail artifacts (B-mode), absence of a seashore sign (M-mode), and presence of lung point sign. These surrogate signs are reported to have a high sensitivity and specificity in diagnosing a pneumothorax [8].

In the Council of Emergency Medicine Residency Directors Academy of Emergency Ultrasound (CORD-AEUS) emergency ultrasound milestones consensus document, assessment for pneumothorax with ultrasound was listed under core skills [9]. Despite extensive research done on the use of thoracic ultrasound to diagnose pneumothorax in different clinical settings, little research has been done to date to develop a training model for pneumothorax [10–13]. To the best of our knowledge, there are no commercially available simulation models to train emergency physicians in ultrasound diagnosis of pneumothorax. The objective of this study was to assess a human cadaver model for training emergency medicine (EM) residents in the ultrasound diagnosis of pneumothorax.

## 2. Methods

### 2.1. Study Design

This was a single-blinded cross-sectional study using a human cadaveric model. The study was approved by the Institutional Review Board. An informed consent was obtained from all the subjects.

### 2.2. Study Setting and Population

This study was conducted at an academic medical center in the Advanced Anatomy Laboratory of the Department of Genetics, Cell Biology, and Anatomy. The primary investigators of this study were two board-certified emergency physicians with expertise in point-of-care ultrasound. Participation in the study was voluntary and included EM residents with no prior experience in thoracic ultrasound. The residents had variable experience in performing FAST ultrasound examinations.

### 2.3. Study Protocol

Three lightly embalmed human cadavers were used in this study. The embalming technique used to prepare the human donors was originally described by Anderson [14] and further modified using glutaraldehyde rather than formaldehyde as described by Wadman et al. [15]. Approximately 8 L of Coinjection of beta factor (Champion Millenium, The Champion Co., Springfield, OH) diluted 1 : 16 with tap water was injected into the carotid artery of the cadaver at a pressure of 500 mm Hg and a flow rate of 400–500 mL/min. The internal jugular vein was opened before injecting beta factor to permit free drainage. Next, approximately 8 L of arterial 24 alpha factor (Champion Millenium, The Champion Co.) diluted 1 : 10 to 1 : 16 with tap water was injected into the carotid artery (pressure of 500 mm Hg and a flow rate of 400–500 mL/min). The embalmed cadaver was stored at 4 degrees Celsius in a plastic bag. This preparation not only preserves tissue texture and color over an extended period of time compared to unembalmed specimens but also reduces the potential for exposure to infectious agents.

**Figure 1:**
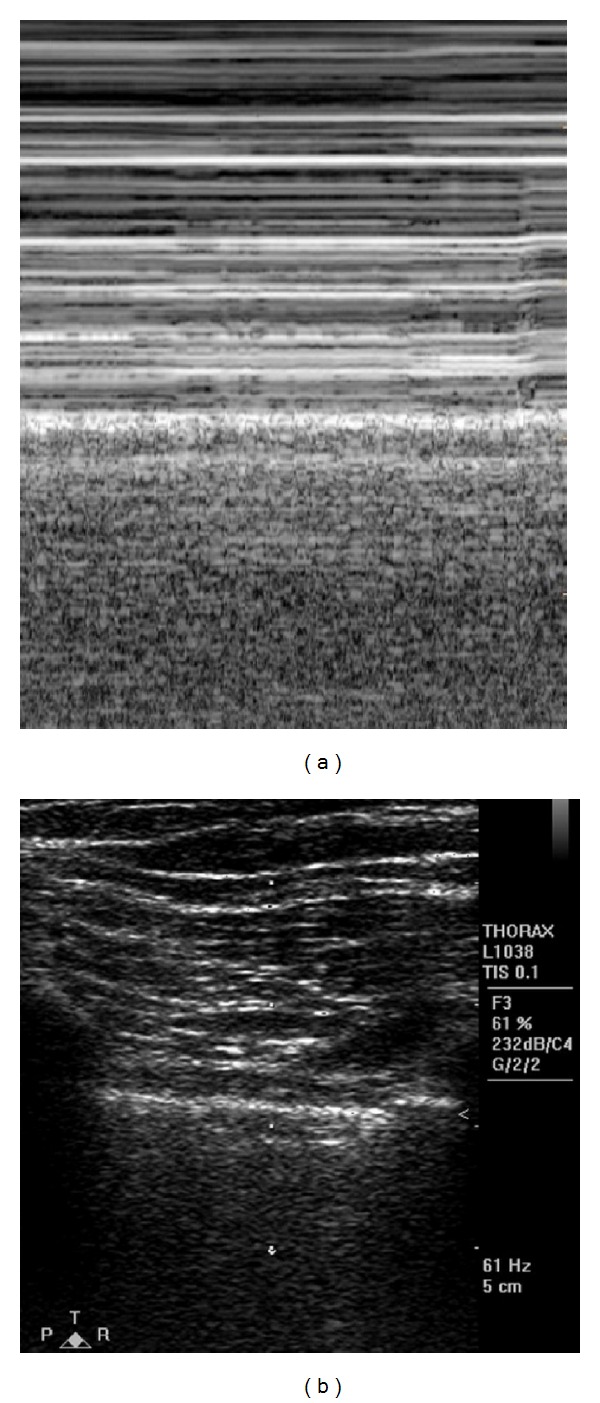
(a) M-mode image showing presence of seashore sign. (b) Corresponding B-mode image showing rib shadows and pleural line.

**Figure 2:**
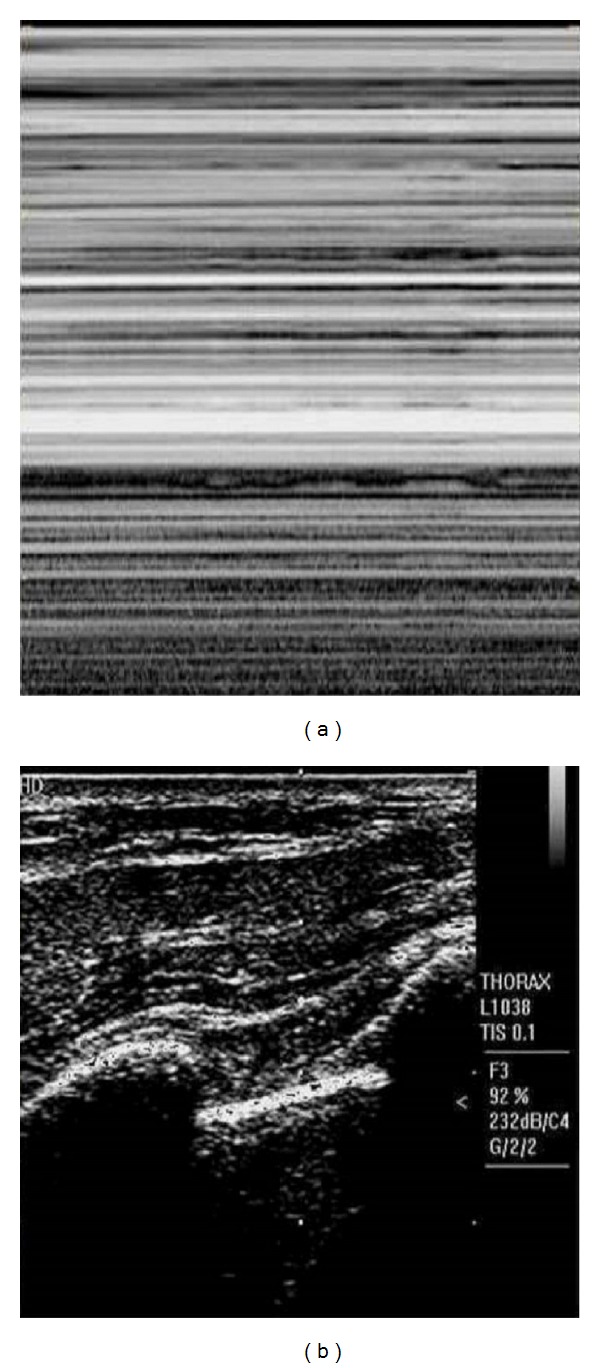
(a) M-mode image showing absence of seashore sign. (b) Corresponding B-mode image showing rib shadows and pleural line.

Cadavers with significant thoracic trauma or significant thoracic pathology were not used. Three “normal lungs” and three lungs altered to model a “pneumothorax” were created. A main stem bronchus intubation or ligation of the main stem bronchus was performed on three lungs to create sonographic signs of pneumothorax. The signs of pneumothorax were the result of absence of ventilation from contralateral main stem bronchus intubation or ligation of the main stem bronchus. A main stem bronchus intubation was performed orotracheally on two cadavers. The absence of lung ventilation on the contralateral side of main stem bronchus intubation was confirmed by observing chest wall movement and auscultation using positive-pressure ventilation with a bag-valve device. On one cadaver, a posterior thoracotomy incision was performed and ribs were spread apart. The underlying structures were dissected to expose the main stem bronchus. The ligation of main stem bronchus was performed and the cadaver was then orotracheally intubated. Absence of lung ventilation on the side of main stem bronchus ligation was confirmed by observing chest wall movement and auscultation during positive- pressure ventilation. With ventilation, the presence or absence of the sliding-lung sign on B-mode ultrasound and sea shore sign on M-mode was also confirmed (Figures 1 and 2). Absence of sliding sign and sea shore sign was defined as a pneumothorax. The cadaver model was pilot tested on three ED attending physicians prior to the study.

**Figure 3:**
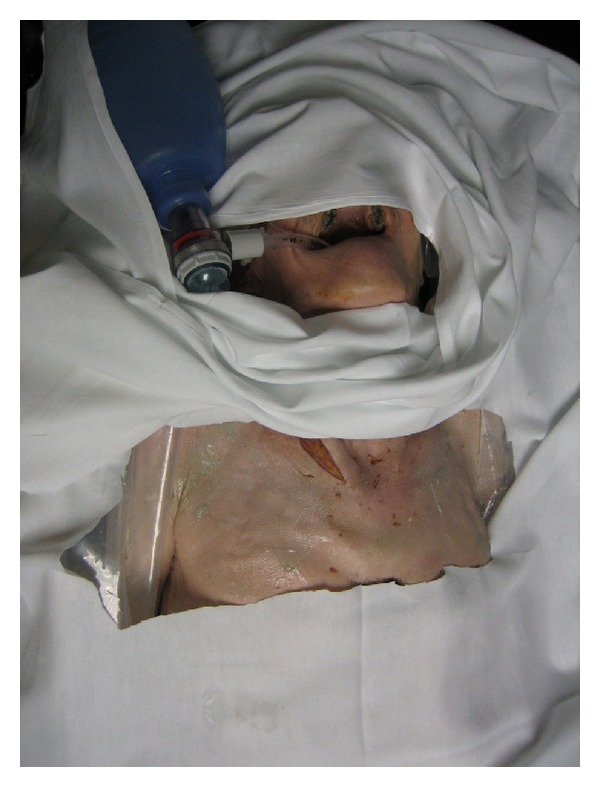
Lightly-embalmed cadaver training model.

**Figure 4:**
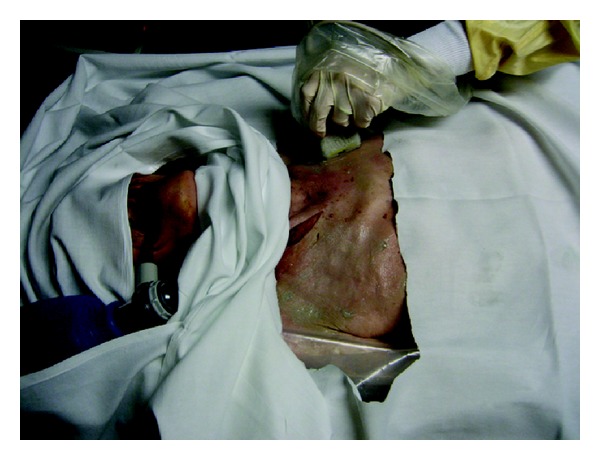
Probe placement on cadaver training model to detect a pneumothorax.

EM residents participated in a 1-hour point-of-care thoracic ultrasound training session which included didactics, image review, and hands-on scanning on the cadavers. Residents were given time to practice on cadavers. The location and the sequence of cadavers were changed after the hands-on session. Each cadaver's head, neck, and chest (except the scanning locations) were draped with a sheet to blind the subjects to endotracheal tube placement, depth, and chest wall movement (Figures 3 and 4). Residents were blinded to the side and number of pneumothoraces, as well as to each other's findings. They obtained images on both sides of the chest along the anterior chest, midclavicular, and midaxillary line during positive-pressure ventilation. A Philips Envisor ultrasound system (Bothell, Washington) with a 12–5 MHz broadband linear transducer was used to obtain real-time images of the hemithorax. The cadavers were ventilated using bag-valve device with the same tidal volume to ensure blinding of the residents. Each resident performed the ultrasound examination and recorded results individually. Residents were allowed to use the M-mode settings and manipulate the other settings of the ultrasound system as desired. They were asked to point out the sonographic findings to the investigators and mark the presence or absence of the sliding-lung sign and sea shore sign on each side of the chest. On the basis of the presence or absence of the sliding-lung sign and seashore sign, a diagnosis of pneumothorax was made by the residents. They were evaluated on their ability to identify the presence or absence of the sliding-lung sign and seashore sign during ventilation of cadavers. Time taken to perform the ultrasound examination (both B-mode and M-mode) was separately recorded. Each hemithorax was reassessed by the investigators in between study participants to verify the fact that the sonographic appearance has not changed. After the ultrasound examination, residents filled out a questionnaire which consisted of items regarding the past ultrasound experience, comfort, and confidence level in the diagnosis of pneumothorax using B-mode and M-mode on the cadaver model.

### 2.4. Outcome Measures

The primary outcome measures were the sensitivity and specificity of residents using ultrasound to distinguish pneumothorax from normal lung on the cadaver model. The secondary outcome measures were confidence and comfort level of EM residents in identifying a pneumothorax.

### 2.5. Data Analysis

The sample size of 45 observations was determined based on producing a 95% confidence interval (CI) with precision of 0.08 when the estimated sensitivity and specificity are 90%. Data were analyzed by using SAS software (Copyright, SAS Institute Inc., Cary, NC, USA). SAS version 9.2 is used for all summary statistics and analyses. Sensitivity and specificity are summarized using descriptive statistics with the associated 95% CIs. Continuous data are presented as means and categorical data were presented as frequencies and percentages with 95% confidence intervals. A chi-square test was used for comparisons. The statistical level of significance used in all analyses was 0.05.

## 3. Results

A total of 84 ultrasound examinations (42-“normal lung” and 42-“pneumothorax”) were performed by 14 EM residents. A sliding-lung sign was accurately identified in 39 scans, and the seashore sign was accurately identified in 34 scans. The sensitivity and specificity for the sliding-lung sign were 93% (95% CI, 85–100%) and 90% (95% CI, 81–99%), respectively. The sensitivity and specificity for the seashore sign were 80% (95% CI, 68–92%) and 83% (95% CI, 72–94%), respectively. The average time to complete the B-mode scan was 33 seconds, and the average time to complete the M-mode scan was 22 seconds.

Ten residents reported that they had performed <50 scans (FAST examinations) and 4 residents had performed 50–100 scans (FAST examinations). Seventy-nine percent (95% CI, 57–100%) reported that they were comfortable using B-mode ultrasound and 64% (95% CI, 39–89%) reported that they were comfortable using M-mode ultrasound to identify a pneumothorax. Seventy-one percent (95% CI, 47–95%) agreed that focused thoracic ultrasound training on human cadaver model is an adequate model for training to diagnose a pneumothorax in a clinical setting. On a scale of 1 to 10, the average confidence level in interpreting the images by residents was 5.8 (95% CI, 4.9–6.7). No statistically significant differences were noted in confidence and comfort level between residents of differing ultrasound experience (*P* = 0.5).

## 4. Discussion

Our study results indicate that human cadaver model can be used effectively for training residents in the ultrasound diagnosis of pneumothorax. To our knowledge, our study is the first one to use human cadavers for thoracic ultrasound training. After a focused thoracic ultrasound training session, the majority of study participants rated the educational experience highly on the questionnaire. Our residents had a high degree of comfort using B-mode ultrasound to assess a pneumothorax. Despite a moderate degree of confidence in interpretation, residents performed well in identifying the surrogate signs of pneumothorax on ultrasound.

Based on our study findings, we believe that lightly embalmed cadavers provide an excellent model for the sonographic appearance of pneumothorax. By preserving the tissue texture and elasticity encountered in clinical setting, lightly embalmed cadavers provide a model that closely simulates residents experience with patients. Unlike commercially available high-fidelity simulators, these cadavers offer a range of anatomic variations and tissue haptics which are crucial for optimal translation of skills learned through practice on experimental models to clinical practice. Use of porcine models for thoracic ultrasound training has been described in the literature [11, 12, 16]. Unfortunately this requires sacrificing a living animal and the anatomical differences between human and pig are problematic. Scanning technique, probe placement, and maneuvers are not identical between human and pig and these factors may affect translation of procedural skills to real-world clinical setting.

The other advantages of human cadaver models include durability. We did not notice any decay of the model or changes in sonographic signs over time. In our experience, lightly embalmed cadaver models can be used for 3 to 4 months for repetitive practice sessions with preserved tissue haptics similar to actual patient experience. The majority of residency training programs use cadavers to teach procedural skills to residents. The techniques we described in this study could be used to create a pneumothorax on the same cadavers for thoracic ultrasound training. Thoracic ultrasound skills can be taught simultaneously with other procedural skills on the same cadavers.

There are several limitations in our study including a small sample size which may limit the conclusions that could be reached. We used a convenience sample of residents which might have introduced selection bias. Although ventilation of the cadaver lungs yielded appropriate sonographic signs in this study, postmortem changes may affect the ultrasound images. We did not investigate other sonographic signs of pneumothorax such as A-line sign, lung point sign, and absence of comet tail artifacts. Lack of cardiac activity is another drawback since the lung pulse sign is caused by transmission of heart beats through the motionless lung. Other limitations include cost of cadaver preparation and storage as well as expertise required for embalming cadavers. This single institution investigation limits the generalizability of our study findings. Our Anatomy Department Faculty was very supportive of our ultrasound program, which may not be the case with other institutions. Additional research with a larger sample is needed to further evaluate our cadaver model to teach sonographic diagnosis of pneumothorax at other institutions.

## 5. Conclusions

Lightly embalmed human cadavers may provide an excellent model for mimicking the sonographic appearance of pneumothorax. Our study results suggest that a human cadaver model can be used to train physicians in ultrasound diagnosis of pneumothorax.
